# Age-related change in muscle strength, muscle mass, and fat mass between the dominant and non-dominant upper limbs

**DOI:** 10.3389/fpubh.2023.1284959

**Published:** 2023-11-23

**Authors:** Jing Pang, Fuyi Tu, Yiwen Han, Enyi Zhang, Yan Zhang, Tiemei Zhang

**Affiliations:** ^1^The Key Laboratory of Geriatrics, Beijing Institute of Geriatrics, Institute of Geriatric Medicine, Chinese Academy of Medical Sciences, Beijing Hospital, National Center of Gerontology of National Health Commission, Beijing, China; ^2^School of Science, Chongqing University of Posts and Telecommunications, Chongqing, China

**Keywords:** upper limbs, muscle strength, lean mass, fat mass, bone mineral content

## Abstract

**Background:**

Any form of physical activity is recommended for the older adults to maintain their physical function; however, the effect of daily activities on muscle function still needs to be investigated. Humans always use one dominant hand to perform tasks, providing a natural situation for research on the effect of daily activities on muscle function.

**Methods:**

Five hundred and twenty-six healthy adults were recruited from the community in Beijing. Muscle strength was assessed using a handgrip dynamometer, lean mass, fat mass, bone area and bone mineral content of upper limbs were assessed using dual-energy X ray-absorptiometry. The results were compared between the dominant and non-dominant upper limbs.

**Results:**

The dominant upper limb had better muscle strength, lean mass, bone area and bone mineral content than the non-dominant side. The difference in muscle strength and lean mass between the two upper limbs decreased with the advanced age. In older age, fat mass of upper limbs increased in men, but not in women.

**Conclusion:**

Daily activities can maintain better muscle function in the dominant upper limb than in the non-dominant side; however, the delaying effect on age-related decline in muscle function was limited.

## Introduction

Sarcopenia is an adverse change in muscle across the whole lifetime, especially in older age. It is defined by the loss of muscle mass, and decreases in muscle strength and physical performance (1, 2). Older adults people with less muscle might have a higher risk of disability. Walking is a basic requirement for independent living, thus most studies have focused on the change of lower limbs with aging (3). There have been relatively fewer studies about the age-related decline in the upper limbs.

The upper limbs play important roles in everyday living, such as feeding, dressing, and grooming, which are critical tasks for independent living. The decline in upper limb strength may impact these activities and reduce quality of life (4, 5). In the Asian Working Group for Sarcopenia 2019 consensus, low muscle strength is defined as handgrip strength <28 kg for men and < 18 kg for women (6). Low handgrip strength is a simple and powerful predictor for future disability (7, 8) and falls (9–11). Similar to lower limb function, upper limb function also involves several physiological domains. A previous study demonstrated that performance in muscle strength, arm stability, dexterity, and coordination of the upper limbs significantly decreased with age (12). To study the upper limbs, handgrip strength is the most popular indicator for muscle function assessment (13). However, muscle strength is only one part of muscle performance, and how other muscle indicators change in the upper limbs with aging are unknown.

In contrast to the lower limbs, people usually have a dominant side when performing tasks using the upper limbs. The dominant side always has greater muscle strength than the non-dominant side (14). Several physical activities, such as doing housework and playing with balls, usually practice only one side of the upper limbs. Therefore, the comparison of muscle performance between the two upper limbs might provide guidance to evaluate the effect of daily activities on muscle function maintenance.

Muscle performance is a loosely defined concept that broadly includes muscle mass, muscle strength, and muscle composition (15). Herein, we compared several muscle indicators between the two upper limbs and observed the age-related changes in them. The results might help us to realize the relationship among daily activities, muscle function and aging, and give us a clue to determine which kind of muscle indicators could be used for early recognition and intervention in motor function decline. This can help us to optimize the intervention strategies for healthy aging.

## Methods

### Subjects

The healthy subjects were volunteers to participant in this study. 538 Chinese adults (526 right-hand dominant and 12 left-hand dominant) aged 25–89 years were recruited from the community of Beijing in 2015. The proportion of left-dominant and right-dominant participants was imbalance; therefore we only analyzed the data of right-hand dominant participants (Table 1). A brief questionnaire, including basic demographic details, physical status, exercise, and medical history information was completed. Subjects who participated in regular strength training of the upper limbs (>1 time/week and > 10 min/time training) were excluded. The subjects’ informed consent was obtained to use their information in this study; and the study was approved by the Ethics Committee of Beijing Hospital (approval No. 2012BJYYEC-052-02).

**Table 1:** Parameters of the dominant and non-dominant upper limbs in muscle performance between men and women (mean ± SD).

Group	Total (*n* = 526)	Men (*n* = 284)	Women (*n* = 242)
Age (years)	62.47 ± 14.36	62.56 ± 14.25	62.37 ± 14.52
Weight (kg)	64.73 ± 10.86	69.91 ± 9.72	58.65 ± 8.78
Height (cm)	164.21 ± 8.06	169.57 ± 5.69	157.92 ± 5.46
BMI (kg/cm^2^)	23.92 ± 3.04	24.28 ± 2.80	23.51 ± 3.26
*Muscle strength (kg)*			
Non-dominant	31.80 ± 9.96	37.33 ± 8.63	25.30 ± 7.08
Dominant	36.72 ± 12.26	42.89 ± 10.62	29.47 ± 9.87
*p***-**Value	<0.001	<0.001	<0.001
Cohen’s *d*	0.88	0.87	0.94
*Bone Area (cm^2^)*			
Non-dominant	187.99 ± 30.93	208.35 ± 23.03	164.09 ± 19.99
Dominant	209.88 ± 36.71	231.23 ± 31.05	184.84 ± 25.22
*p***-**Value	<0.001	<0.001	<0.001
Cohen’s *d*	1.19	1.20	1.18
*Bone Mineral Content (g)*			
Non-dominant	141.97 ± 43.51	170.7 ± 33.18	108.25 ± 26.78
Dominant	153.18 ± 44.26	184.21 ± 32.48	116.76 ± 23.72
*p***-**Value	<0.001	<0.001	<0.001
Cohen’s *d*	0.47	0.50	0.45
*Lean Mass (g)*			
Non-dominant	2139.54 ± 614.96	2564.47 ± 463.60	1640.87 ± 330.08
Dominant	2293.31 ± 684.15	2777.51 ± 508.55	1725.08 ± 339.74
*p***-**Value	<0.001	<0.001	<0.001
Cohen’s *d*	0.57	0.75	0.36
*Fat Mass (g)*			
Non-dominant	1275.74 ± 416.87	1196.39 ± 354.94	1368.87 ± 463.13
Dominant	1309.93 ± 387.88	1189.41 ± 315.32	1451.38 ± 416.87
*p***-**Value	<0.001	0.466	<0.001
Cohen’s *d*	0.18	0.04	0.38

*p*-Value: differences between the dominant and the non-dominant upper limbs. Cohen’s d: the difference between the means of the dominant and the non-dominant upper limbs divided by the standard deviation.

### Muscle strength

Handgrip strength (HS) was assessed in each hand using a JAMAR digital dynamometer (Sammons Preston, Chicago, IL, United States), which was measured twice for each hand and the higher value was used in the analysis.

### Muscle mass and muscle composition

All the participants completed the dual-energy X ray-absorptiometry (DEXA, Hologic QDR 4500A, Hologic) measurement in Beijing hospital. A standardized procedure for body composition detection and QDR software analysis were used. From DEXA scans, lean mass, fat mass, bone area and bone mineral content (BMC) of two upper limbs were evaluated.

### Statistical analysis

Descriptive statistics were used to characterize the demographics and the measured variables of the subjects. All values are shown as the mean ± SD. The differences between the two upper limbs were compared using a paired *t*-test. A sample size of 29 achieves 90% power to detect a mean of paired differences of 3.0 with an estimated standard deviation of differences of 5.0 and with a significance level (alpha) of 0.05 using a two-sided paired t-test. The statistical analyses were carried out using SPSS 21.0 (IBM Corp., Armonk, NY, United States), and *p* < 0.05 was considered statistically significant.

For analysis the age-related patterns of muscle performance in two upper limbs, we employed the restricted cubic spline (RCS) to flexibly model and visualize the relationships between age and each indicator. RCS fitted a smooth and continuous curve for each indicator across the range of ages, allowing for cubic form changes in function at specific age points - 45, 55, 65 and 75 years - referred to as knot points. The curves obtained by RCS reflect the overall age-related changes of each indicator, and we further identified the change points at which abrupt changes occur in each indicator during ageing based on the fitted RCS curves. The segmented regression is a common choice for change point detection (16, 17). Given an initial guess for the change point, the segment regression fitted a piecewise linear function and updated the change point to minimize the gap between the two fitted intersecting lines. The process iterated until convergence is reached, ultimately yielding the optimal change point. The left and right slopes are calculated together with the estimated change point. The analyses of this part were performed using R (Version 4.3.1).

## Results

### The difference in muscle performance between the two upper limbs

All the muscle performance indicators (muscle strength, lean mass, fat mass, bone area and bone mineral content) were exhibited significantly differences between the two upper limbs (*p* < 0.05, Table 1). The muscle strength, lean mass, bone area, and bone mineral content of the dominant upper limb were much higher than those of the non-dominant side both in male and female participants (*p* < 0.05, Table 1). In women, the dominant upper limb had more fat mass than the non-dominant one (p < 0.05); however, there was no difference in fat mass between the two sides in men.

### Comparison of muscle performance between the two upper limbs among age groups

In females, the dominant upper limb exhibited higher muscle strength, lean mass, fat mass, bone area, and bone mineral content than the non-dominant upper limb in each age group (Table 2). In males, the muscle strength, lean mass and bone area showed significant differences between the two upper limbs in each age group; however, there was no difference of bone mineral content and fat mass between the two sides in older group. We calculated the value of the dominant upper limb minus the non-dominant upper limb (D-value). The difference in muscle strength and lean mass between the two upper limbs decreased significantly with age both in male and female participants. Compared with those in 25–59 years old group, D-value for muscle strength in 75+ years old group decreased by 54% for males and 55% for females; D-value for lean mass in 75+ years old group decreased by 58% for males and 59% for females.

**Table 2:** Parameters of the dominant and non-dominant upper limbs in muscle performance within different age groups (mean ± SD).

Group	Men			Women		
	25–59 years	60–74 years	75+ years	25–59 years	60–74 years	75+ years
*n*	103	122	59	89	100	53
Height (cm)	171.42 ± 5.42	168.93 ± 5.80	167.66 ± 5.04	159.13 ± 5.36	158.31 ± 4.74	155.13 ± 5.99
Weight (kg)	71.91 ± 10.43	69.64 ± 9.60	67.00 ± 7.85	58.57 ± 8.68	59.53 ± 9.01	57.13 ± 8.45
BMI (kg/cm^2^)	24.42 ± 2.84	24.37 ± 2.83	23.84 ± 2.68	23.13 ± 3.27	23.75 ± 3.44	23.70 ± 2.85
*Muscle strength (kg)*						
Non-dominant	42.50 ± 8.48	36.28 ± 6.98	30.49 ± 6.13	29.24 ± 6.92	24.61 ± 6.10	20.00 ± 4.91
Dominant	49.75 ± 10.20	41.51 ± 8.50	33.80 ± 6.70	34.72 ± 10.23	28.52 ± 8.77	22.45 ± 5.43
*p***-**Value	<0.001	<0.001	<0.001	<0.001	<0.001	<0.001
Cohen’s *d*	0.98	0.97	0.60	1.15	0.92	0.68
*Bone area (cm^2^)*						
Non-dominant	207.59 ± 23.49	210.30 ± 22.65	205.66 ± 23.04	168.78 ± 21.32	164.86 ± 17.64	154.77 ± 19.05
Dominant	231.46 ± 33.49	234.53 ± 28.79	224.00 ± 30.47	188.48 ± 25.33	184.94 ± 24.99	178.52 ± 24.68
*p***-**Value	<0.001	<0.001	<0.001	<0.001	<0.001	<0.001
Cohen’s *d*	1.12	1.47	0.92	1.01	1.24	1.46
*Bone mineral content (g)*						
Non-dominant	176.61 ± 27.06	168.88 ± 27.19	164.14 ± 49.27	118.33 ± 19.44	108.21 ± 31.48	91.40 ± 18.30
Dominant	192.66 ± 33.06	183.81 ± 28.94	170.31 ± 33.96	127.86 ± 19.36	114.84 ± 21.84	101.76 ± 24.85
*p***-**Value	<0.001	<0.001	0.182	<0.001	0.013	<0.001
Cohen’s *d*	0.59	0.68	0.18	0.94	0.25	0.79
*Lean mass (g)*						
Non-dominant	2664.80 ± 527.32	2584.63 ± 425.97	2347.61 ± 338.48	1619.62 ± 370.59	1697.64 ± 285.27	1569.42 ± 324.73
Dominant	2965.60 ± 520.36	2765.62 ± 488.69	2473.75 ± 361.20	1748.28 ± 364.33	1758.98 ± 325.86	1622.15 ± 306.95
*p***-**Value	<0.001	<0.001	<0.001	<0.001	<0.001	0.017
Cohen’s *d*	0.86	0.76	0.62	0.40	0.40	0.34
*Fat mass (g)*						
Non-dominant	1184.89 ± 388.73	1200.59 ± 347.64	1207.77 ± 310.52	1344.98 ± 501.49	1391.80 ± 475.64	1365.73 ± 368.48
Dominant	1148.46 ± 322.15	1202.69 ± 318.99	1233.43 ± 291.57	1407.08 ± 469.03	1486.92 ± 395.03	1458.70 ± 360.81
*p***-**Value	0.031	0.887	0.153	0.029	<0.001	<0.001
Cohen’s *d*	0.22	0.01	0.19	0.23	0.46	0.62

*p*-Value: differences between the dominant and the non-dominant upper limbs; Cohen’s d: the difference between the means of the dominant and the non-dominant upper limbs divided by the standard deviation.

### Comparison of age-related changes in muscle performance between the two upper limbs

**Figure 1:**
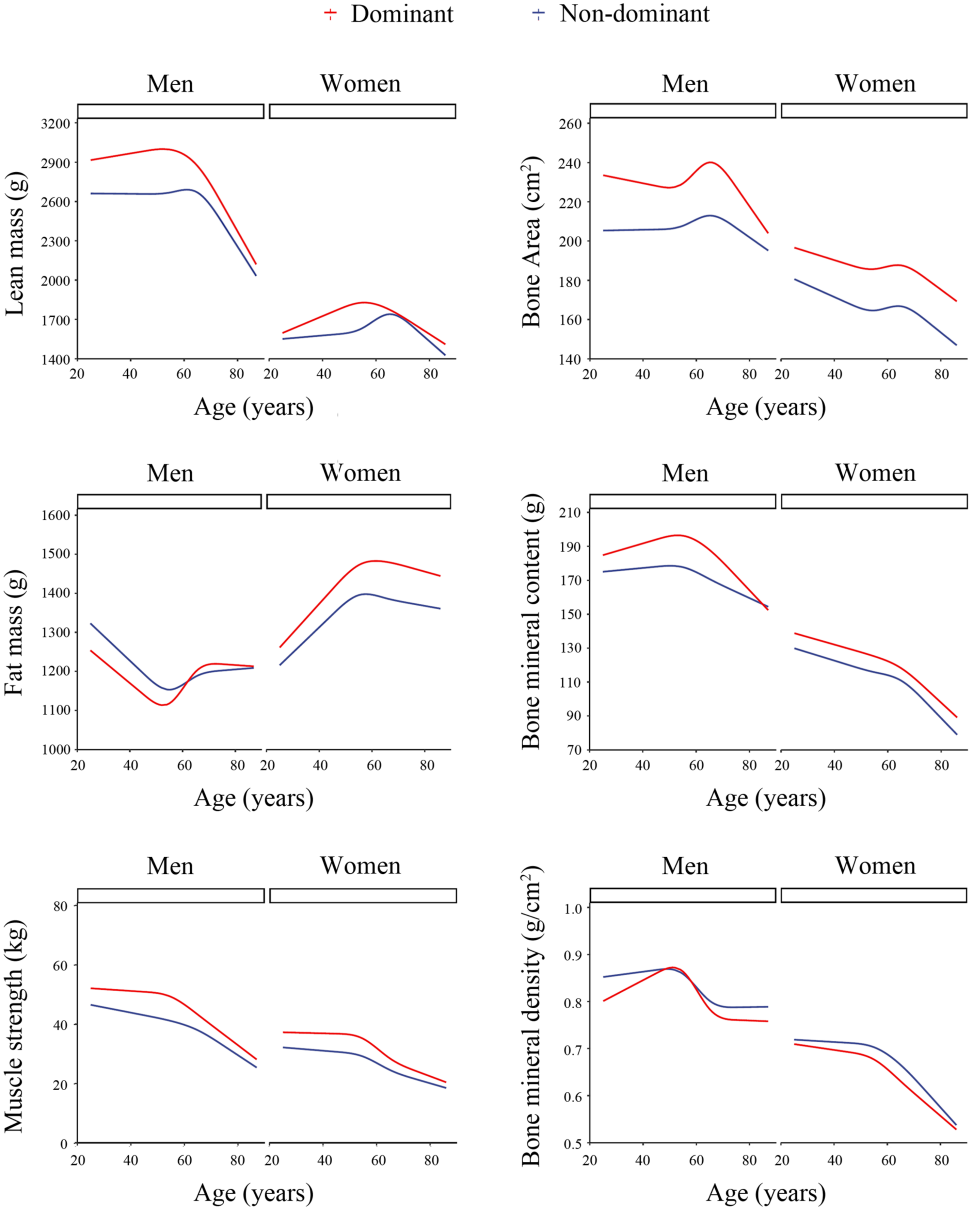
Age-related changes in muscle performance indicators between the two upper limbs. The curves obtained by the restricted cubic spline analysis. The red line represented the change of the dominant upper limb; the blue line represented the change of the non-dominant upper limb.

**Figure 2:**
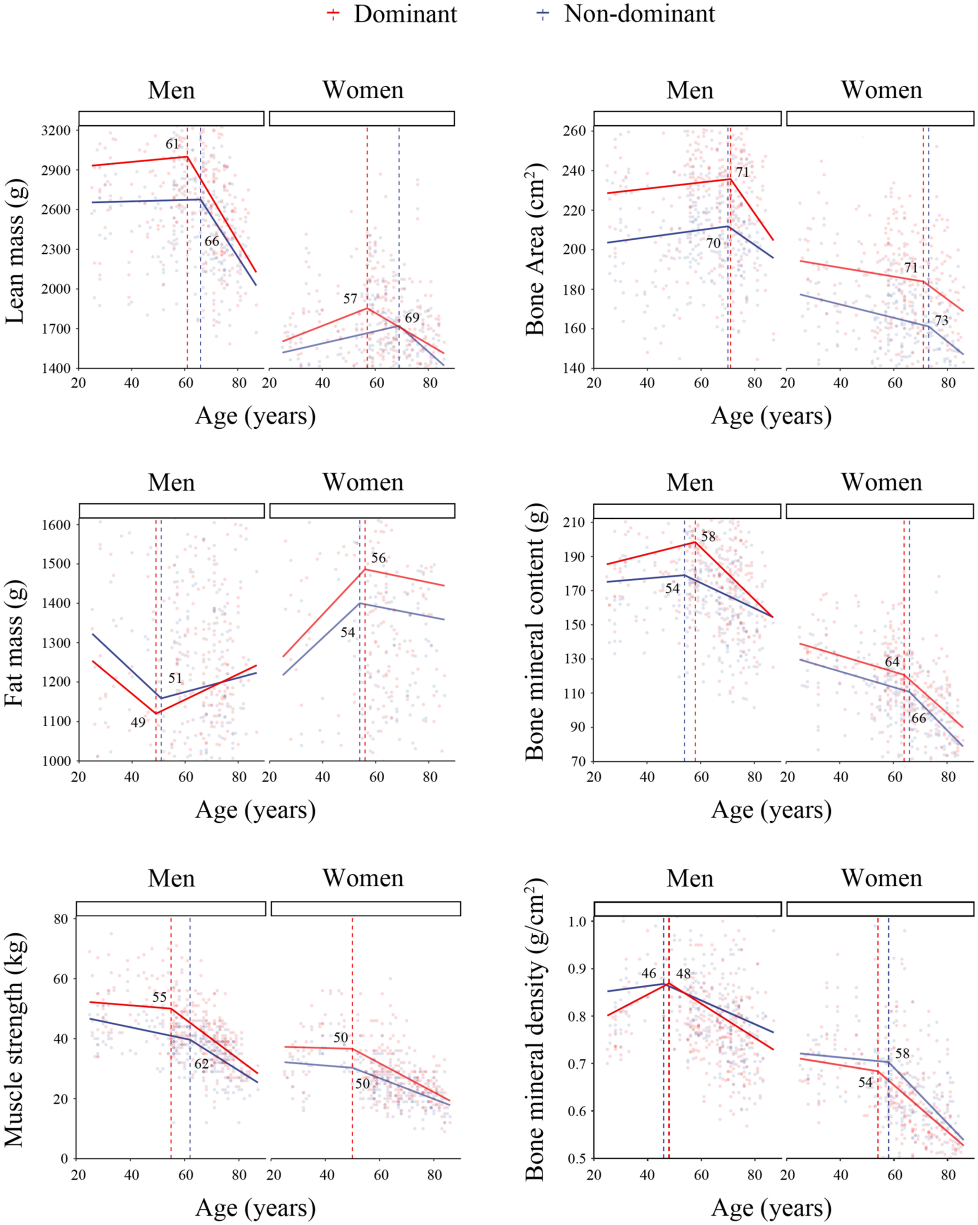
The cutoff points of muscle performance indicators changed with age. The age at the cutoff point was marked. The red line represented the change of the dominant upper limb; the blue line represented the change of the non-dominant upper limb.

We observed the trend of muscle performance indicators changed with age (Figure 1), analyzed the cutoff point of each indicator (Figure 2) and calculated the slope of them (Table 3). After 50 years old, the slope of muscle strength and lean mass in the dominant upper limb declined more significantly than those in the non-dominant upper limb both in males and females participants. The change of fat mass showed a sexual difference. After 50 years old, the fat mass of both upper limbs increased significantly in males, but decreased in females. In males, the slope of bone mineral content changed more significantly in the dominant upper limb than that in the non-dominant upper limb after 65 years old. In females, the age-related trend of bone mineral content was similar between the two upper limbs.

**Table 3:** The slope of age-related changes in muscle performance indicators.

Indicators	Before cutoff point		After cutoff point	
	Men (*n* = 284)	Women (*n* = 242)	Men (*n* = 284)	Women (*n* = 242)
*Muscle strength*				
Non-dominant	−0.1896	−0.0742	−0.5711	−0.3465
Dominant	−0.0725	−0.0249	−0.6764	−0.4822
*Bone area*				
Non-dominant	0.184	−0.3386	−0.9462	−1.0905
Dominant	0.155	−0.2262	−1.9421	−1.0017
*Bone mineral content*				
Non-dominant	0.1348	−0.4622	−0.7447	−1.5933
Dominant	0.394	−0.4717	−1.5254	−1.3958
*Lean mass*				
Non-dominant	0.5416	4.5967	−30.978	−17.69
Dominant	1.8802	7.8551	−33.619	−11.782
*Fat mass*				
Non-dominant	−6.3086	6.3029	1.8025	−1.3025
Dominant	−5.5937	7.1818	3.2365	−1.3951

## Discussion

Physical activity is important for physical function maintenance. People always have one dominant hand for carrying out daily tasks. The dominant hand is usually involved in more physical activity than the non-dominant hand, which might lead to different muscle performance between them. In this study, we compared several muscle performance indicators of the two upper limbs and found that the dominant upper limb maintained the better muscle performance than the non-dominant side, but the difference between the two sides decreased with the advanced age.

In this study, the handgrip strength of the dominant upper limb declined faster than that of the non-dominant upper limb in older age. Consistent with this, men had stronger handgrip strength than women, but the strength of men declined faster than that of women during aging (18, 19). Daily activities can increase muscle strength, but cannot prevent muscle aging. There may be two ways for muscle function maintenance: (1) doing more activities from young age to reserve high intrinsic ability; (2) engaging in more activities in old age for anti-aging.

Muscle strength and muscle mass are two important indicators for motor function assessment (1, 2); however, which indicator is more sensitive for sarcopenia early recognition still need to be investigated. In this study, both muscle strength and lean mass of upper limbs showed significant differences between the two sides and declined with age. In addition, we observed that muscle strength began to decline significantly at age 55 in men and age 50 in women, which was earlier than lean mass declined at age 61 in men and age 57 in women. This result supported the European consensus on Sarcopenia diagnosis revised in 2019, in which muscle strength detection was modified as an early screening indicator for muscle mass loss (2). Previous studies also found that physical intervention affected muscle strength, but had little effect on muscle mass (20, 21). According to these data, muscle strength might be more sensitive to reflect the change in muscle function than muscle mass.

The change of muscle composition, the loss of muscle mass and the infiltration of fat into muscle, was a main indicator for muscle quality evaluation and a cause of muscle function decline (22, 23). Here, the age-related change in fat mass showed a difference between the two sexes. In men, the dominant upper limb had little fat mass than the non-dominant side in young age, and the fat mass of both upper limbs increased after 50 years old. Contrary to men, the dominant upper limb had more fat mass than the non-dominant side from young to old in women. This sex difference might have been caused by hormone effect. The decrease of testosterone, which is a potent anabolic factor promoting muscle protein synthesis and muscular regeneration, may induce muscle composition changed (24).

Bone loss is a major risk for fall-related injury in older adults. Interestingly, the dominant upper limb had more bone area and bone mineral content than the non-dominant upper limb. Bone mineral density is a common indicator for bone health assessment, which is calculated by dividing bone mineral content by bone area. Previous studies had proved that high-intensity physical activity is an effective contributor to bone mineral density (25). Inconsistent with this, the bone mineral density of two upper limbs was similar in this study. These results suggested that daily activities might promote large bone area development in adolescent and maintained it into old age, but were not enough to increase bone mineral density.

This was the first study to use the difference in daily activities between the two upper limbs to determine the relationship among muscle performance, physical activity, and aging. In daily living, the dominant upper limb always completes more daily activities than the non-dominant side. Does this kind of physical activity slow the age-related decrease in muscle function? Our data showed that daily activities can promote high muscle function reserve, but cannot prevent muscle function decline with aging. In addition, the interaction of person and environment has been a focus of attention. Environmental factors, such as living arrangements, financial status, and electronics, may influence the lifestyle of older adults and have potential confounding impacts on muscle performance (26, 27). In this study, data on the amounts of physical activity and other influence factors were not investigated, thus more detailed analysis was missing. Further study may focus on the quantitative analysis between physical activity and muscle function maintenance during the aging process.

## Conclusion

**Figure 3:**
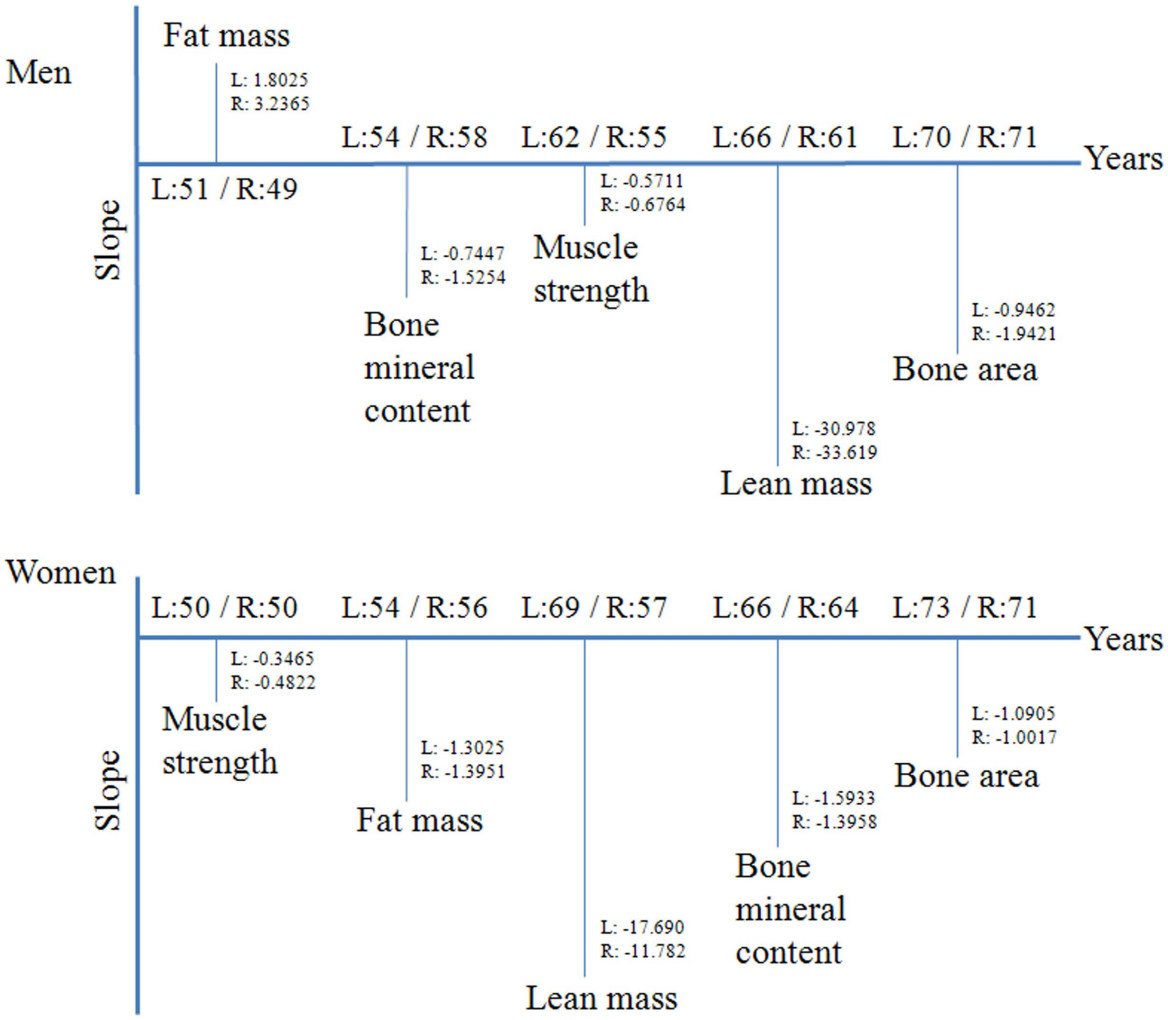
The key points of muscle performance indicators changed with age. The horizontal line marked the age of each indicator changed significantly. The vertical line marked the slope of each indicator changed after the cutoff point. L: the non-dominant upper limb; R: the dominant upper limb.

Muscle strength, lean mass, fat mass, bone area and bone mineral content were obviously different between the two upper limbs, and showed different aging patterns (Figure 3). In older adults, despite more regular use, the dominant hand’s muscle and bone quality was not protected against ageing. Additional exercise should be recommended for older adults to delay motor function decline and promote healthy aging. Comparison of the age-related trends of muscle performance indicators, fat mass increased earliest in men, and muscle strength declined first in women. The detection of muscle strength and fat mass may be useful for early recognition and intervention of motor function decline. Overall, as compared with the performance of the non-dominant upper limb, the dominant upper limb showed better muscle performance and bone quality from young to old. The long-term beneficial effect of daily activities on muscle and bone health was verified. Engaging in more physical activities at a younger age should be recommended to maintain independent living ability and decrease the occurrence of sarcopenia and falls later in life.

## Data availability statement

The original contributions presented in the study are included in the article/supplementary material, further inquiries can be directed to the corresponding authors.

## Ethics statement

The studies involving humans were approved by the Ethics Committee of Beijing Hospital. The studies were conducted in accordance with the local legislation and institutional requirements. Written informed consent for participation in this study was provided by the participants' legal guardians/next of kin.

## Author contributions

JP: Funding acquisition, Investigation, Project administration, Writing – original draft, Writing – review & editing. FT: Data curation, Writing – original draft. YH: Investigation, Writing – original draft. EZ: Investigation, Writing – original draft. YZ: Investigation, Writing – original draft. TZ: Funding acquisition, Project administration, Writing – review & editing.
